# #MedTwitter: An Open Source Medical Community

**DOI:** 10.15694/mep.2021.000019.1

**Published:** 2021-01-21

**Authors:** Ethan Fieger, Kristen Fadel

**Affiliations:** 1 Virginia Commonwealth University

**Keywords:** Twitter, Social Media, Medical Education, Covid-19, Networking, Hashtag, #MedTwitter

## Abstract

This article was migrated. The article was not marked as recommended.

As social media platforms such as Twitter become increasingly utilized by physicians, students, and researchers for professional use, it is important to further the discussion about the role of these sites in medicine. Here, we focus on the hashtag #MedTwitter, which consists of an online community of researchers, practitioners, students, and professionals who have created an open source, decentralized forum for information share, medical education, and professional networking. #MedTwitter also provides a space for publications to be shared, promoted, and discussed, which has proven particularly useful in the setting of the Covid-19 pandemic. #MedTwitter comes with its challenges, potential for abuse, and the spread of misinformation. Thus, further research and discussion will be critical in exploring the future of #MedTwitter and social media in healthcare.

## Introduction

Twitter: “It’s what’s happening.” Since its creation in 2006, the social media and microblogging platform has represented a novel, occasionally controversial ecosystem of news, information, and trending hashtags. With over 330 million users estimated in 2019, 145 million of which were considered daily users, Twitter has solidified itself as an important source of information-and dialogue-worldwide ( Lin, 2019). While the general public uses Twitter to circulate news, keep up with their favorite celebrities, and share their thoughts, the scientific community has adopted Twitter in a unique capacity. Almost 25% of physicians surveyed in one study use social media on a daily basis for medical purposes ( Eysenbach, 2011). The question of whether there is a future for social media in medicine finds at least one emergent answer on Twitter. This article intends to share the ways #MedTwitter has proven useful to the medical community, while also inviting discussion of the potential for misuse in this unregulated information exchange.

The hashtag #MedTwitter reveals an active, vibrant online community of researchers, practitioners, and students who have effectively created an open source forum for information share, medical education, and professional networking. Most compelling about #MedTwitter is the lack of hierarchy and pretense, a welcome contrast to the realities of medical education and professional life. #MedTwitter is accessible to medical students, residents, fellows, attendings, and researchers who want to share recently published articles or collaborate, unbound by the status quo and other barriers to development at their institutions. This community offers a completely open-source environment that democratizes medical information. It welcomes novices to the field, as well as experts hoping to extend their impact through retweets and follower growth.

## Clinical Research and Practice

Beyond its largely social functionalities, #MedTwitter is used to share medical discoveries, clinical findings, clinical algorithms, and published treatments of disease among digital users in the medical community. This value has become particularly evident amidst the Covid-19 pandemic. Since the onset of the pandemic, there has been much uncertainty surrounding the virus, including the nature of transmission, the symptomatology, the clinical course, and potential treatments. As information surfaces daily and treatment protocols continue to evolve, #MedTwitter allows healthcare providers to keep apace with a rapid news cycle. #MedTwitter facilitates the free exchange of Covid-19 research findings, anecdotal experiences of caring for infected patients, and updates regarding clinical trials. The value of the #MedTwitter platform in such an unprecedented time is the easy access to this plethora of vital and timely information, all found in one place and supplemented by the opportunity for dialogue among the medical community. These benefits extend beyond the pandemic to many acute and chronic medical conditions. For example, active users of #MedTwitter may stumble across publications highlighting the potentially successful use of gene editing as a treatment for sickle cell disease. Additionally, this space provides the opportunity for authors to share their own publications in hopes of reaching a wider audience, and allows the audience to directly reach these authors with questions and comments. One study showed that the number of times an article is tweeted may potentially increase the number of article citations ( McGowan *et al.,* 2011).

## Networking and Professional Development

The networking opportunities provided by #MedTwitter are vast and allow connections at every strata of the medical field. This is especially beneficial for medical students, who often struggle to reach senior leaders, even at their own institutions. #MedTwitter allows students to connect directly to physicians, residency program directors, researchers, and more. These connections expand far beyond one’s own institution, allowing students to ask questions about particular residency programs; ask for timely advice regarding rotations or applications; and further explore specialties via experienced clinicians in those fields. In fact, many residency programs have created accounts that allow them to interact directly with students. Networking through #MedTwitter also provides the opportunity to find research and leadership opportunities, seek out mentors, access webinars, and get involved in different organizations across the country. Medical students are also able to interact with one another and share study resources and experiences, as well as collaborate on projects. These networking opportunities also exist for providers, who are able to connect with and learn from others within and outside of their immediate circle or institution. #MedTwitter allows them the space to share anecdotal experiences that may help other clinicians, as well as pose scientific or clinical questions to a vast medical community with ranging expertise. Additionally, providers are able to stay up to date regarding changes in their field, and in medicine as a whole.

## Medical Education

Correspondingly, #MedTwitter has become an open source for medical education and advice at every level. In fact, several tributary hashtags have been created, often used in conjunction with #MedTwitter, to direct users to educational material. These include (but are not limited to) #MedEd, #MedTweetorials, and #TipsForNewDocs. These hashtags are often included in “threads,” which are a series of connected tweets, often delivering one message. These threads serve to teach a medical concept, walk through diagnostic schemas, or share personal experiences and counsel. Several examples include “Approach to giving IV fluids,” “ICU for interns,” and “Schema video for elevated Troponin.” The concise tweets shared through these hashtags vary in complexity, and are likely beneficial at several levels of training.

## Potential Challenges

It must be noted that the same decentralizing attributes that make #MedTwitter valuable also welcome potential danger. #MedTwitter is an unregulated, unfiltered, and unreviewed body of information, meant to be social in nature. There are no post standards, validation processes, or promises of accuracy for any information posted to the feed. Though much of the information on #MedTwitter involves published articles from reputable journals, there is still potential for abuse: namely, violation of physician-patient confidentiality, the creation of fraudulent accounts, the promotion of harmful agendas, or the spread of outdated or mis-information. Another important consideration is the potential for information on #MedTwitter to be interpreted as medical professionals offering advice. This presents both ethical and medical legal concerns, as the lay reader may make interpretations without full knowledge of the subject. Many users specify “no medical advice” or “all opinions are their own” in their account biographies, to insulate themselves from this potential issue. These issues mirror larger, systemic ills perpetuated by social media giants and their co-optation of mainstream news and politics. Most profiles are unvalidated by the platform’s regulatory “blue check,” and some are even anonymous. Certainly, this is a reminder that peer review, validation, and institutional associations are vital to science and medicine. The potential for misuse and abuse should be considered by anyone viewing #MedTwitter content. This invites the question of how #MedTwitter and social media as a whole should be utilized responsibly, and of whether the added value of #MedTwitter exceeds the potential harm.

## Conclusion

#MedTwitter has proven to be a potentially valuable resource for medical students, clinicians, and researchers to network and share knowledge, though not without significant potential risks. Many students and professionals would argue that there is a place for #MedTwitter, even if it relies on well-intentioned users who approach it with a healthy level of caution and skepticism. Only further discussion and research will uncover the true role of social media in medicine, ultimately deciding whether its influence will expand, fade, or transition into a more regulated space.

## Take Home Messages

• Social media platforms such as Twitter are rapidly growing in popularity among physicians for professional use
• #MedTwitter is an online community composed of medical providers, researchers and students
• #MedTwitter is a decentralized forum for information exchange, research promotion, networking, and medical education
• #MedTwitter is an unregulated and unreviewed platform that presents significant potential for abuse and the spread of misinformation
• Further discussion and research is needed to explore the future of social media in medicine

## Notes On Contributors

**Ethan Fieger** is a medical student at the Virginia Commonwealth University School of Medicine. He received his Bachelors of Arts degree in biology from the University of Virginia. His current research interests include the use social media in medicine, nonalcoholic fatty liver disease, diabetes management, obesity, and the ketogenic diet.

**Kristen Fadel** is a medical student at the Virginia Commonwealth University School of Medicine. She received her Bachelors of Science degree in biology from James Madison University. Her current research interests include the use social media in medicine, nonalcoholic fatty liver disease, diabetes management, obesity, and the ketogenic diet.
