# Supplementary material for: #MedTwitter: An Open Source Medical Community
Source: MedEdPublish (2016). 2021 Sep 22;10:19. Originally published 2021 Jan 21. [Version 2] doi: 10.15694/mep.2021.000019.2 (PMC10939582; doi:10.15694/mep.2021.000019.2)
Supplement: Peer review history

# Reviewer report: Reviewer response for version 2

**DOI:** 10.21956/mep.20208.r31287

**Published:** 2021-10-09

**Authors:** Ken Masters

**Affiliations:** 1 Sultan Qaboos University

This review has been migrated. The reviewer awarded 4 stars out of 5 This is a nice (albeit short) opinion piece highlighting the strengths of #MedTwitter. It is a timely piece also, because Twitter appears to have gained a reputation for poor postings, and #MedTwitter and related areas appear to be shining contrary examples.I do also like the fact that the authors have balanced their enthusiasm with a note of caution, for, indeed, there are the dangers as outlined by the authors.A good and useful piece, especially useful for the novice.

Reviewer Expertise:

NA

No decision status is available

# Reviewer report: Reviewer response for version 2

**DOI:** 10.21956/mep.20208.r31286

**Published:** 2021-10-07

**Authors:** Julie Hunt

**Affiliations:** 1 Lincoln Memorial University

This review has been migrated. The reviewer awarded 4 stars out of 5 This opinion piece discusses the use of Twitter, and specifically the Twitter hashtag #MedTwitter, in sharing and disseminating research and clinical findings, and networking among all levels of medical professionals. The two students who wrote it are to be commended on researching and writing the piece. This version 2 appears superior to version 1 in its inclusion of more and newer references to support the assertions made in the article. The authors do a good job of offering the drawbacks of Twitter use along with the benefits. Ultimately, only time may tell how social media use and medical knowledge dissemination interact in the future.

Reviewer Expertise:

NA

No decision status is available

# Reviewer report: Reviewer response for version 1

**DOI:** 10.21956/mep.19010.r27277

**Published:** 2021-02-28

**Authors:** Megan Anakin

**Affiliations:** 1 University of Otago

This review has been migrated. The reviewer awarded 2 stars out of 5 I read your article with interest because I occasionally participate in using Twitter to discuss medical education research with colleagues. I would like to build on Subha Ramani’s encouraging comments by making several suggestions to enhance your article. In the second sentence of the introductory paragraph, the number of Twitter users is supported by a reference to a commercial website ‘Oberlo’ from 2019, please consider supporting this claim with an up-to-date scholarly reference. The Eysenbach 2011 reference does not support the claim made in the sentence beginning: “Almost 25% of physicians …”. The McGowan et al. 2012 should be used cautiously here because findings come from a 2011 study with a 29% response rate from oncologists and primary care physicians in the US only. Claims about the use of Twitter should be supported by more recent and varied references to inform the international readers of this article. In the ‘Clinical Research and Practice’ section, please provide a reference to support the claim in the third sentence of the paragraph. Please note that the McGowan et al. 2012 reference provided to support the claim about the number of tweets and article citations is incorrect. Please consider revising this statement to more accurately reflect the study’s focus and findings by Eysenbach 2011. A more recent reference would better support this point.In the ‘Potential Challenges’ section, please consider providing references to support the claims made in the second and third sentences, and for the sentences beginning: “Many users specify …” and “Most profiles are …”I would be very happy to read and review a revised version of this article.

Reviewer Expertise:

NA

No decision status is available

# Reviewer report: Reviewer response for version 1

**DOI:** 10.21956/mep.19010.r27278

**Published:** 2021-01-21

**Authors:** Subha Ramani

**Affiliations:** 1 Harvard Medical School, Brigham and Women's Hospital

This review has been migrated. The reviewer awarded 3 stars out of 5 I enjoyed reading this summary about application and value of dedicated Twitter sites to clinical practitioners, teachers and learners and health professionals interested in joining a community. I like the way the authors categorized the areas- practice, education, community. The challenges that they anticipate and inform us about are real. The one thing to consider is that social media users are not homogenous, they vary in expertise and degree of use. Experts in social media should continue to consider how evidence-informed information reaches their audiences especially those who use the platform in limited ways and want to get the most accurate information possible, This is a well written summary of a particular Twitter hashtag. While I appreciate the information in this paper, I am still wondering how these potential challenges can be overcome.

Reviewer Expertise:

NA

No decision status is available
